# Methodological Gaps in Left Atrial Function Assessment by 2D Speckle Tracking Echocardiography

**DOI:** 10.5935/abc.20150144

**Published:** 2015-12

**Authors:** Roxana Cristina Rimbaş, Raluca Elena Dulgheru, Dragoş Vinereanu

**Affiliations:** 1 University and Emergency Hospital, Bucharest - Romania; 2 University of Medicine and Pharmacy Carol Davila, Bucharest - Romania; 3 "Victor Babeş" National Institute of Pathology, Bucharest - Romania

**Keywords:** Atrial Function, Left, Heart Atria / abnormalities, Echocardiography, Diagnostic Imaging

## Abstract

The assessment of left atrial (LA) function is used in various cardiovascular diseases. LA plays a complementary role in cardiac performance by modulating left ventricular (LV) function. Transthoracic two-dimensional (2D) phasic volumes and Doppler echocardiography can measure LA function non-invasively. However, evaluation of LA deformation derived from 2D speckle tracking echocardiography (STE) is a new feasible and promising approach for assessment of LA mechanics. These parameters are able to detect subclinical LA dysfunction in different pathological condition. Normal ranges for LA deformation and cut-off values to diagnose LA dysfunction with different diseases have been reported, but data are still conflicting, probably because of some methodological and technical issues. This review highlights the importance of an unique standardized technique to assess the LA phasic functions by STE, and discusses recent studies on the most important clinical applications of this technique.

## Introduction

Left atrial (LA) remodeling was described as an important prognostic marker in different diseases, such as heart failure, myocardial infarction, hypertrophic cardiomyopathy, and atrial fibrillation^1-5^.Although the assessment of LA function can be obtained by conventional 2D echocardiography, Doppler analysis of transmitral and pulmonary vein flows, and tissue Doppler measurement of LA myocardial velocities, its detailed quantification remains challenging.

2D Speckle tracking echocardiography (2DSTE) is a feasible technique for the assessment of myocardial LA deformation^6-9^. Its quantification may provide more insights into the LA mechanics^10-12^. However, normal ranges for LA strain and strain rate (SR) are still debatable^5,13-16^.

The aim of our review is to discuss the main advantages and limitations of assessing LA deformation by 2DSTE.

We will refer to atrial physiology in order to discuss the main functions of the LA, and how STE can be used to assess them. Since the vast majority of the studies looking to LA deformation used EchoPac (GE Medical Systems), we will exemplify our comments by using images derived from this software, which was previously validated for the analysis of LA strain with high feasibility and good agreement^6-8,17^.

### Left atrial physiology

The LA function contributes to LV filling by all three components: a reservoir (40%), a passive conduit (35%), and a pump component (25%)^18,19^. Prolonged ventricular relaxation leads to a decrease in conduit function, while the reservoir and pump functions increase. As diastolic dysfunction progresses, the passive conduit function increases, while the reservoir and active pump functions decrease significantly^18^.

LA contractile function depends on preload, afterload, intrinsic contractility, atrial electrical activation, and electromechanical coupling. Propagation of electrical impulse occurs throught interatrial connections in the subepicardium of the LA^17^. This results in LA atrial activation from the interatrial septum to the inferior, anterior, and lateral LA walls during sinus rhythm. Changes in these pathways may prolong or abolish interatrial conduction, and create a substrate for atrial arrhthmyias^17^.

### Left atrial phasic functions assessed by STE

Currently, strain and SR parameters derived from 2DSTE allow us to identify all components of LA function^8,16^. However, this technique has some limitations. It is frame-dependent and cannot be used in patients whose 2D image quality is suboptimal. Thus, STE needs high quality-scale images, and requires a learning curve. Otherwise, it is a very promising tool for the assessment of regional and global LA function^6-8,13^.

Longitudinal LA strain and SR parameters can assess atrial function in several pathological conditions, such as mitral valve disease, supraventricular arrhythmias, hypertension, heart failure, and cardiomyopathies. However, the lack of standardization is an important limitation to the widespread use of these parameters in routine clinical practice.

Consequently, normal values for all these 3 components of the LA function are highly required.

The atria and ventricles move in opposite directions during the cardiac cycle, so the atrial myocardium lengthens during ventricular systole (positive strain), while the ventricular myocardium shortens during ventricular systole (negative strain). This creates a mirror image for S/SR curves of the LA and LV.

Cameli et al^8^ described a 12-segments model for the analysis of LA strain, using 4- and 2-chamber apical views. Other studies proposed a 15-segments model for a complete evaluation of the LA strain, using 4-, 2-, and 3- chamber views^6,7,18^. This variability of the model used is one of the technical factors that might create different normal values for strain and SR parameters, and also different cut-off values in pathological conditions.

It is already known that there are regional differences in the LA segmental function during atrial contraction and relaxation,with the posterior wall having the lowest strain, probably due to the fact that its motion is limited by attachment of the pulmonary veins^6,7^, and the inferior wall exhibiting the highest deformation, attributable to its greater thickness. Therefore, ignoring the posterior wall by using a 12-segments model, might overestimate the global S and SR parameters. Similarly, the atrial reservoir strain is greater in the apical 2C than in the 4C view, since the 4C view incorporates two areas in which atrial strain is low (the interatrial septum and the area of the pulmonary veins).

**Figure 1:**
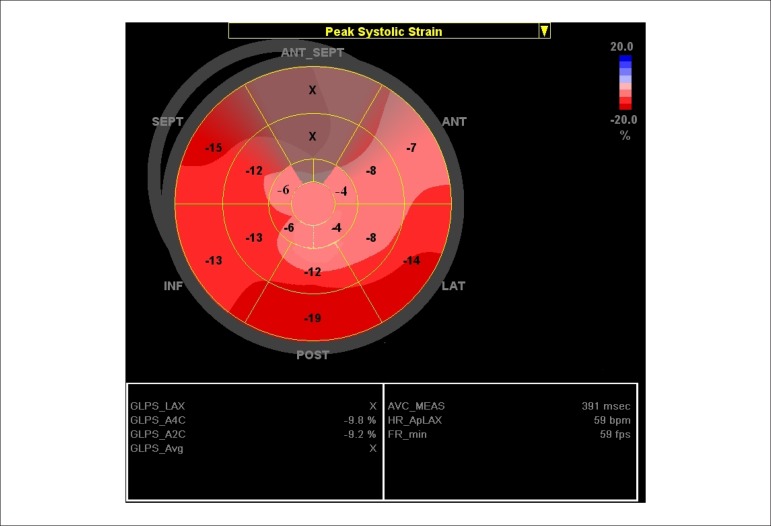
"Bull’s eye" view of the longitudinal 2D strain of the left atrial contraction in a normal subject. It is coded in red because it represents the atrial strain of the pump function. Basal values are higher than medial values, and further reduced in the center, which represents the atrial roof (light red). Antero-lateral values are lower than infero-posterior values. Values of the antero-septal wall were excluded, because they correspond to the ascending aorta.

During atrial contraction and relaxation, a deformation gradient is observed from all views, with higher strain in the atrio-ventricular junction and lower strain in the atrial roof, because the atrial roof is fixed to the mediastinum (Figure 1).

In our opinion, full assessment of the LA function by 2DSTE must include apical 2-, 4-, and 3- chamber views, optimized for the visualization of the LA.

The frame rate should be set between 60 and 80 frames per second^16,17^. To trace the region of interest (ROI) in the discontinuity of the left atrial wall corresponding to pulmonary veins, the direction of LA endocardial and epicardial surfaces at the junction with these structures should be extrapolated^6-8^.

Before processing, a cine loop preview confirms that the internal line follows the LA endocardium throughout the cardiac cycle. Manual adjustments will be made when tracking of the LA endocardium is unsatisfactory.

LA segments with inadequate image quality must be rejected. We suggest excluding from the analysis the subjects with more than one non-acceptable segment per chamber. Tracing the LA cavity just before atrial contraction, when it is smaller, often eliminates myocardial wall dropout in the interatrial septum and the pulmonary veins and, therefore, improves tracking. Tracking the more hyperdynamic parts of the LA, such as the annular lateral, inferior, and inferior-posterior regions, can be challenging. Extending the LA endocardial trace a little apically below the mitral annulus and adjusting the post-processing settings to better define the LA in this area might be helpful.

**Figure 2:**
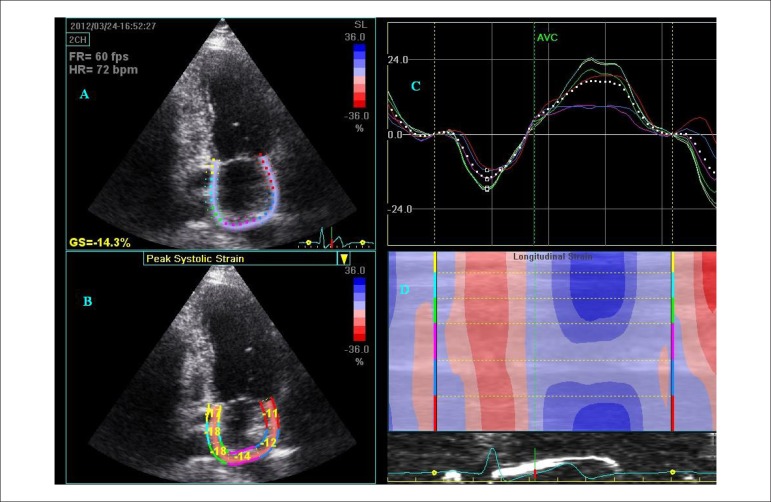
Quad view of the longitudinal LA strain by 2DSTE. 2DSTE of the left atrium (LA) from the 2C view, depicting the region of interest (ROI) created by the STE software (panel A), and the corresponding regional strain values of the atrial function (panel B). In panel C, LA strain curves for each of the 6 segments are analyzed. The dashed curve represents the mean LA longitudinal strain. Reference point was placed at the onset of the P-wave. During the period in which the atrium acts as a reservoir atrial strain increases, reaching a peak just before mitral valve opening. During the conduit function, atrial strain decreases, with a plateau during diastasis, and a negative peak at the end of atrial contraction. In panel D, curved M-mode shows that atrial roof positive strain is lower (in light blue) than the other walls (in dark blue).

Longitudinal left atrial S and SR parameters must be assessed as the average of 6 segmental values per each view (Figure 2). The final S/SR values will be the average of the values obtained for each apical view, excluding the three segments of the antero-septal wall from the 3-chamber view, corresponding to the ascending aorta.

**Figure 3:**
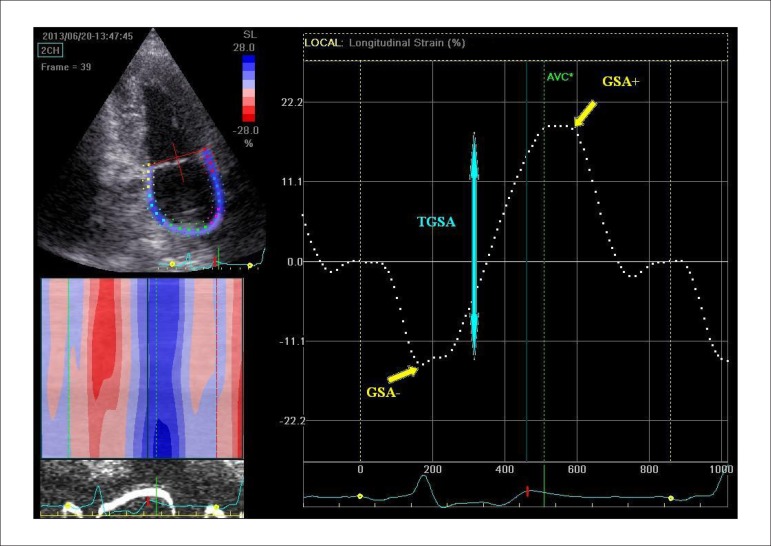
Measurement of parameters of left atrial longitudinal strain by 2DSTE. 2C view depicting the region of interest and curved M-mode created by the software (left), and the corresponding global left atrial longitudinal strain (right); AVC: Aortic valve closure. The reference point was placed at the onset of the P wave, allowing measurement of the negative global strain at maximal atrial contraction (GSA-) (pump function), positive global strain at mitral valve opening (GSA+) (conduit function), and also sum of GSA- and GSA+ (TGSA) (reservoir function).

The strain curve evaluated by 2DSTE must follow the LA physiology. During the LA reservoir function, corresponding to the LV isovolumic contraction, ejection and isovolumic relaxation, LA strain increases, achieving the highest peak just before mitral valve opening. During the conduit function, LA strain decreases and achieves a negative peak at the end of the LA contraction (Figure 3). Subsequently, during the diastasis, both the S and SR profiles are flat, demonstrating that no LA wall deformation occurs in the late phase of the conduit function^7,12,16^. Using global longitudinal S and SR curves, active, passive, and reservoir function can be defined as follows:

1. Active (pump) function:
  - Negative global strain as a difference between the strain at pre-atrial contraction (after A vawe) and strain just before mitral valve closure (MVC) (GSA-) (Figure 3)^6,7^;
  - Late diastolic global strain rate (GSRL) (Figure 4)^5,7,12^.
    **Figure 4:**
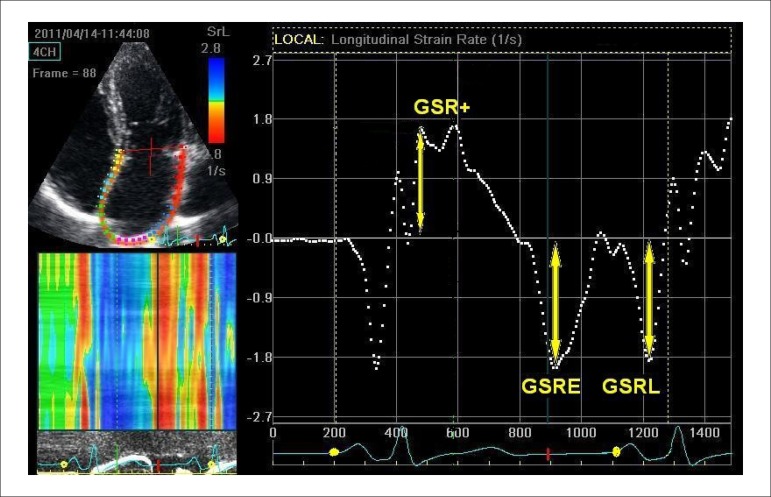
Measurement of parameters of left atrial longitudinal strain rate by 2DSTE. 4C view depicting the region of interest created by the software (left), and the corresponding left atrial global longitudinal strain rate (right). GSR+, first positive global strain rate at the beginning of left ventricular systole; TGSR+, time from the P wave onset to peak positive strain rate; GSRE, early diastolic strain rate; GSRL, late diastolic strain rate.
2. Passive (conduit) function:
  - Positive global strain at MVO (GSA+) (Figure 3)^6,7^;
  - Early negative diastolic global strain rate (GSRE) (Figure 4)^5,7,12^.
3. Reservoir function:
  - Sum of GSA- and GSA+ (TGSA) (Figure 2)^6,7^;
  - First positive global strain rate at the beginning of LV systole (GSR+) (Figure 4)^6,7^.

However, different studies used different methodologies, based on different reference points from the ECG (R-wave or P-wave), for the generation of the strain curve, which might generate different normal values^6-9,12,16^. Saraiva et al^7^, in a normal population, used the P-wave for the generation of the strain curves and found that GSA+ was 21.4 ± 6.7%, TGSA was 35.6 ± 7.9%, while GSA- was -14.2±3.3%. Sun et al^5^, in a similar population, using the R-wave as the reference point, reported completely different values for TGSA+, of 46.8 ± 7.7%^5^. Moreover, due to an upward translation of the strain curve with the R-wave method, they found a positive strain of 19.6 ± 4.2%, interpreted as atrial contraction. The normal values for SR parameters were similar between studies^5,7,8^.

Taking into account atrial physiology, we can easily understand that studies using the R-wave ignore the real active pump function (negative peak), and create a positive strain for this function, inconsistent with the real physiological changes^5,9,12,16^. In contrast to the assessment of LV strain, in which the R-wave from the ECG is used as a reference point, we considered that the use of the P-wave enables the negative global LA strain, which corresponds to the real LA contractile function.

**Figure 5:**
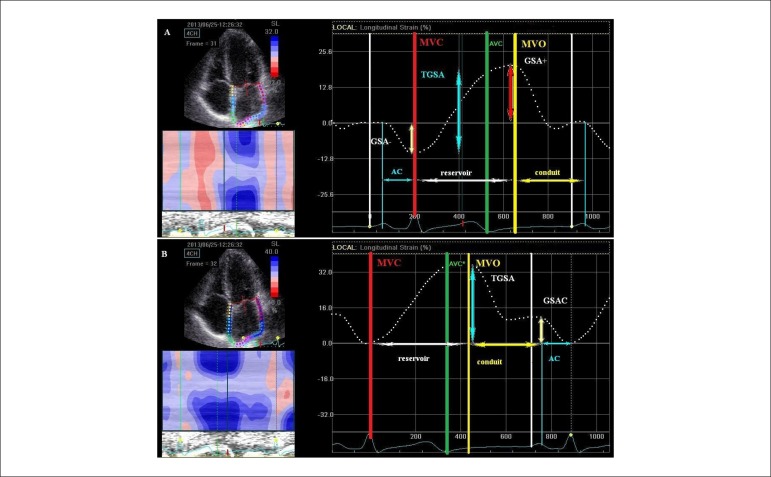
Comparison between the P-wave and the R-wave methods, for LA phasic function. 4C view depicting the region of interest (ROI) (left), and the corresponding LA strain curves (right). The dashed curves represent the mean global atrial longitudinal strains along the cardiac cycle. The reference point was placed at the onset of the P-wave (panel A), and at the R-wave (panel B). MVC: Mitral valve closure; AVC: Aortic valve closure; MVO: Mitral valve opening. Panel A - Measurement of the negative global strain at atrial contraction (GSA-), the positive global strain at MVO (GSA+), and the total strain (TGSA) as a sum between GSA- and GSA+. The pump (AC), reservoir, and conduit functions are depicted. In the left inferior panel there is a clear delineation of the LA pump (red) and reservoir function (blue), with curved M-mode profile. Panel B - Measurement of the total positive global strain at MVO (TGSA) (reservoir), and late positive global strain (GSAC) at the atrial contraction. There is a positive strain for atrial pump function with this method. The conduit function is defined as a difference between TGSA and GSAC. In the left inferior panel there is no delineation at all for the LA contraction and reservoir function (all blue), with curved M-mode profile.

Our experience showed that the R-wave method, by comparison with the P-wave method, provided a non physiological positive value for the active LA function, whereas conduit and reservoir functions (GSA+, TGSA) were significantly overestimated (Figure 5)^20^. These findings suggest that the difference between methods does not consist of a simple upward translation of the strain curve, as previously suggested by Cameli et al^9,12^.

Guidelines recommend the R-wave as a temporal landmark for the LV strain analysis, in order to correctly generate maximal negative strain during the contraction phase. Because after LA contraction the length of the LA is smaller than before contraction, LA contraction strain has to have a negative value. This reflects better the true principles of strain, in which not only the magnitude that represents the strain is important, but also the direction of deformation. We suggest that using P-wave as a reference point might estimate correctly all LA functions^20^.

To standardize LA deformation, other settings, such as proper gain and ROI, should be considered. Firstly, low gain settings artificially eliminate anatomic structures. Alternatively, with excess gain, there is a decrease in resolution. In our experience, increasing gain from minimum to maximum overestimates all LA functions.

Intermediary changes did not have significant impact on active and conduit functions, but they did on reservoir function^20^. This is very important, since many studies focused on reservoir function and its correlation with LV systolic and diastolic functions^21-24^. Secondly, increasing the ROI width decreases values of LA deformation, probably related to the contamination by surrounding structures. Taking into account the LA anatomy, higher ROIs could be used by mistake only if the initial or the postprocessing gain is very high. Because the LA walls are very thin, the minimum ROI should be used^20,21^.The potential difficulty of accurately obtaining a region of interest close enough to the effective shape of the LA, and the risk of contamination by signal components arising from structures surrounding the LA, should be considered also^20-24^.

In conclusion, we suggest that a medium gain and a minimum ROI should be used as the best choice for a standard assessment of LA deformation.

### Clinical applications of the parameters of left atrial deformation

#### General population

LA size has been shown to be a prognostic marker for adverse cardiovascular events in the general population^25-28^. While some studies emphasize the role of both LA volume index (LAVi) and LV diastolic dysfunction as independent predictors of cardiovascular events^29,30^, others doubt the ability of LAVi to predict all cause mortality, independently of the degree of LV diastolic dysfunction^31^. More recently, LA emptying fraction (LAEF) was associated independently with mortality^32^, in a general population based study, and with development of atrial fibrillation (AF) or flutter in subjects ≥ 65 years^33^. Other studies have suggested that LA pump function is also able to identify subjects at higher cardiovascular risk in the population^34,35^, and that minimum LAV may be an important prognostic marker^36,37^. In order to identify the incremental value of LA deformation analysis by 2DSTE as a cardiovascular risk marker, compared with LAVi or LAEF, Cameli et al^38^ evaluated prospectively 312 adults older than 50 years. They showed that global positive strain, using the R-wave method and a 12 segments model of LA, is a strong and independent predictor of cardiovascular events, superior to the conventional parameters of LA analysis^38^.

#### Atrial fibrillation

**Figure 6:**
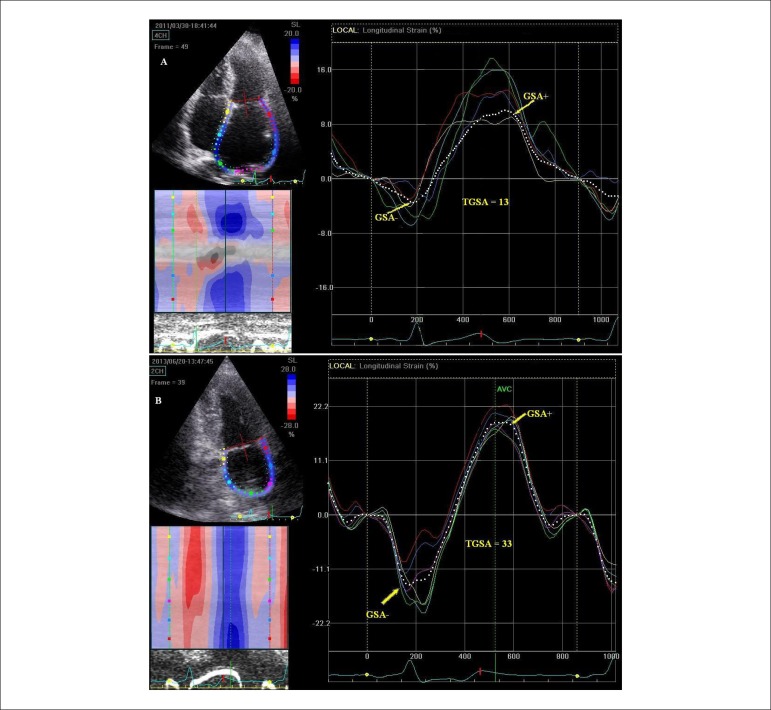
Comparison between left atrial strain in a patient with reccurent atrial fibrillation (panel A) and in a normal subject (panel B). Averaged strain (dotted line) is markedly reduced in the patient with reccurent atrial fibrillation during the pump function (GSA-) (-3% vs. -15%), during the conduit function (GSA+) (9% vs. 18%), and during the reservoir function (TGSA) (13% vs. 33%). There is also a complete dyssynchrony of contraction and relaxation between left atrial segments in the patient with atrial fibrillation versus the normal subject.

Patients with AF have both electrical and morphological LA remodeling. Interstitial fibrosis is one of the landmark morphological changes in patients with AF, and extensive LA fibrosis was associated with impairment of LA function. LA deformation parameters are reduced in patients with non-valvular AF (Figure 6) as compared with normal subjects^39,40^. An inverse correlation was shown between the degree of LA fibrosis, as assessed by MRI, and LA strain and SR, as assessed by vector velocity imaging^41^.

Different studies demonstrated that LA strain predicts the risk of cardiovascular events, or success in restoring sinus rhythm following electrical cardioversion or ablation procedures, in patients with AF. It also predicts the risk of AF recurrence after successful cardioversion^40,42,43^. Thus, in the study of Saha et al^39^, TGSA and total LAEF were reduced, and TGSA was the only index associated with greater odds of a CHADS2 score ≥ 2. Furthermore, LA reservoir strain was incremental to the CHADS2 score in predicting death or hospitalization^39^. Another study showed a reduced GSA- and GSA+ in patients with paroxysmal AF and a CHADS2 score ≤ 1 before their index stroke, by comparison with age and sex matched controls, with paroxysmal AF and no history of stroke. Moreover, GSA- was associated significantly with stroke. These results suggest that LA strain might help the decision for oral anticoagulation in this group of patients^44^.

Shih et al. showed that LA strain during atrial filling and SR during reservoir phase were decreased in patients with AF and stroke, and were associated independently with stroke^45^. Another study showed that although GSA+ was not predictive of AF recurrence in patients who needed cardioversion, the change in peak positive LA strain was significantly higher in subjects who maintained sinus rhythm^46^. The lack of predictive power may be related only to the small sample size. More recently, abnormalities of the timing of atrial deformation showed to predict recurrence of AF after cardioversionm^47,48^. Thus, in patients referred for cardioversion for AF the standard deviation of the time-to-peak strain, using a six segments model of the LA, was an independent predictor of AF recurrence^47^. Similar results have been published after catheter ablation procedures for AF^49^.

#### Cardiomyopathies

**Figure 7:**
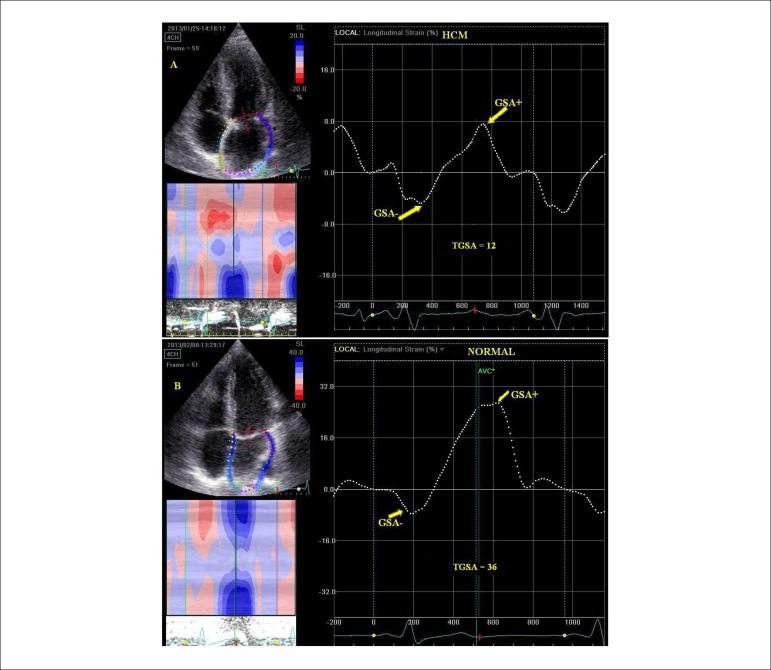
Comparison between left atrial strain in a patient with hypertrophic cardiomyopathy (panel A) and in a normal subject (panel B). Averaged strain is markedly reduced in the patient with hypertrophic cardiomyopathy during the pump function (GSA-) (-4% vs. -10%), during the conduit phase (GSA+) (8% vs. 26%), and during the reservoir phase (TGSA) (12% vs. 36%).

LA strain is reduced in hypertrophic cardiomyopathy (HCM) by comparison to healthy controls (Figure 7), but also compared to patients with secondary LV hypertrophy due to hypertension^22,24^. Moreover, in another study it was suggested that LA strain might have an additive value over conventional parameters, such as LAVi, E/A and E/E' ratio, in differentiating HCM from other types of hypertrophy, with a cut-off value of -10.8% for the pump function^22^. Meanwhile, Rosca et al^2^ showed that LA pump function, evaluated only from the SR curves, is an independent determinant of heart failure symptoms in patients with HCM.

In patients with cardiac amyloidosis, LA dysfunction is also common. In one study using tissue Doppler, peak LA systolic strain and SR were lower in patients with cardiac amyloidosis than in patients with LV diastolic dysfunction of other causes, suggesting that tissue Doppler can be used to detect subtle changes in LA function in these patients^50^. Another study using STE confirmed that LA dysfunction is a common component of amyloidosis, even in the absence of the traditional echocardiographic features. Thus, GSRL and GSA- were significantly lower in amyloidosis compared with the control group, suggesting that assessment of LA deformation is able to detect subtle differences in LA function, not recognized by most conventional parameters. Therefore, it appears that amyloidosis affects LA function above the dysfunction secondary to LV diastolic dysfunction^51^.

In patients with dilated cardiomyopathy (DCM), LA function assessed by STE was severely altered in idiopathic, by comparison with ischemic DCM. In a study on 314 patients, peak systolic LA strain was significantly reduced in idiopathic DCM as opposed to ischemic DCM^11^. However, this study used the R-wave method for the generation of the strain curves, and what they defined as "peak systolic LA strain" was in fact the LA reservoir function (TGSA). Another recent study investigated the importance of LA functional reserve, during dobutamine stress echocardiography (DSE), in patients with depressed LV systolic function^52^. They concluded that the assessment of LA reservoir and passive emptying function during DSE provides important incremental value over standard clinical and echocardiographic parameters to predict cardiovascular events in DCM, since a decreased LA functional reserve was associated with a higher cardiovascular event rate^52^. In another study, in patients with heart failure, TGSA correlated well with the pulmonary capillary wedge pressure (r = -0.81, p < 0.0001), providing a better estimation of LV filling pressure (AUC = 0.93) than E/E' ratio^12^.

**Figure 8:**
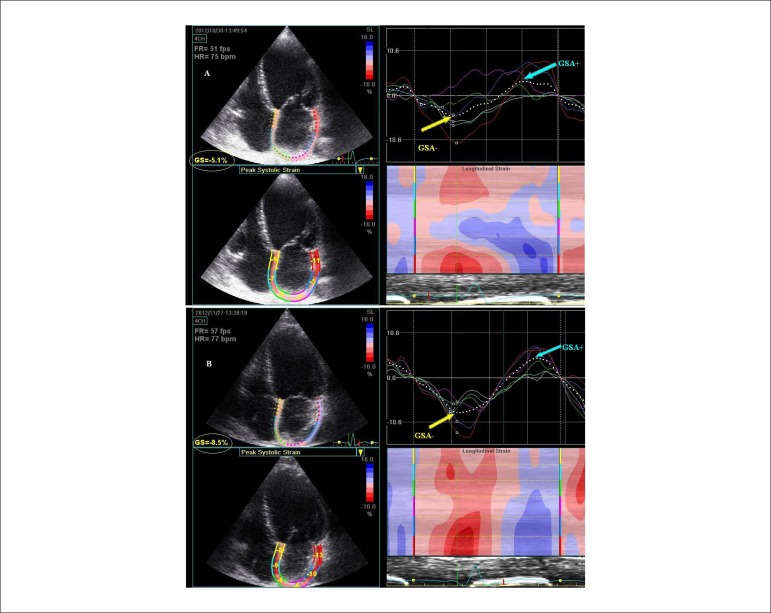
Comparison between left atrial strain in a patient with idiopathic dilated cardiomyopathy, before (panel A) and after (panel B) cardiac resynchronization therapy (CRT). Averaged strain is markedly reduced before than after CRT during the pump function (GSA-) (-5.1% vs. -9%), during the conduit function (GSA+) (2.4% vs. 5%), and also during the reservoir function (TGSA) (7.5% vs. 14%). A significant improvement of the atrial synchrony for both pump and reservoir functions was observed after CRT (panel B), compared to a complete disorganized pattern of strain before CRT (panel A).

LA strain analysis by STE might reveal relevant information in patients with DCM, candidates for cardiac resynchronization therapy (CRT). One study including 90 patients with DCM of either idiopathic or ischemic etiology confirmed that LA systolic function (GSA-) is considerably more impaired in patients with idiopathic than ischemic DCM. Furthermore, CRT responders with ischemic DCM were more likely to have an improvement of LA function after resynchronization (Figure 8). In fact, the only independent determinants of LA functional recovery after CRT were positive response to CRT and the ischemic etiology of DCM^53^. Another study using tissue Doppler showed that in patients with heart failure and CRT, atrial strain was higher in the right atrium, interatrial septum, and left atrium in atrial-sensed compared to atrial-paced mode^54^. This study emphasized that, despite no difference in intraventricular dyssynchrony, patients with atrial-sensed mode had significantly lower atrial dyssynchrony that contributed to a better LV performance after CRT.

#### Ischemic heart disease

LA dysfunction is also common after acute myocardial infarction. In a study on 320 patients evaluated by STE 48 hours after admission for acute myocardial infarction, LA reservoir strain (TGSA) and maximum LAVi were independent predictors of all-cause mortality, re-infarction, and re-hospitalization for chronic heart failure, after adjustment for clinical and other echocardiographic parameters^55^. On contrary, in a study on 843 patients with myocardial infarction, TGSA measured within 48 hours after hospitalization was significantly associated with the composite outcome of heart failure and death, but failed to predict this outcome after adjustment (for age, global longitudinal LV strain, and maximum LAVi). This study suggested that LA strain in these patients is dependent on global LV longitudinal strain and LA size and, therefore, the added prognostic value of LA reservoir function in patients with impaired LV longitudinal function is questionable^56^.

#### Valvular heart disease

LA enlargement and impaired LA function, resulting from volume or pressure overload, is frequent in chronic mitral regurgitation (MR) and aortic stenosis (AS).

LA size proved to be a good predictor of outcome in primary MR: a LA diameter more than 55 mm was associated with a lower 8-year survival rate, while a LA volume more than 60 ml/m^2^ was associated with an increased mortality and cardiac events (AF and heart failure). However, few data are published regarding LA function, as assessed by STE, and its prognostic role in primary MR. A recent study on 121 patients with severe MR reported significant LA reservoir and pump dysfunction, which were more pronounced in patients already having an indication for surgery. Of all indices of LA function, the LA reservoir strain had the highest accuracy to identify patients with indication for mitral valve surgery. Moreover, after mitral valve surgery, patients with LA reservoir strain ≤ 24% showed worse survival after a median follow up of 6.4 years, regardless of the symptomatic status before surgery^57^. This emphasizes once more the importance of a correct assessment of the LA reservoir strain.

Preserved atrial pump function is important for maintenance of cardiac output in patients with severe aortic stenosis. In patients with severe AS, all atrial functions (reservoir, conduit, and pump) were impaired, by comparison with matched controls^58^. As expected, LA reservoir dysfunction was related to LV filling pressures, while LA conduit dysfunction depended on the degree of impaired LV relaxation^58^. Another recent study investigated the role of LA function, assessed by STE, as a predictor of postoperative AF in severe patients with AS undergoing conventional surgery. GSRL was the only independent predictor of postoperative AF, suggesting its role in risk stratification of patients with severe AS^59^.

## Conclusions

The assessment of LA deformation by 2D speckle tracking echocardiography may represent a rapid and easy-to-perform technique to explore LA function. These new parameters of atrial function are more sensitive than traditional indices of atrial function, and could be incorporated into the routine assessment of various heart diseases, such as atrial fibrillation, hypertrophic and dilated cardiomyopathies, ischaemic heart disease, and valvular valve disease. We suggest that methodological standardization is essential in order to introduce LA deformation analysis into the clinical practice. In order to define the normal values and the cut-off values for diagnosis and prognosis in different diseases, we suggest to use the P-wave method for the generation of the strain curve. This method allows a complete evaluation of all LA functions: pump, passive conduit, and reservoir. Gain should be set in the mid range, and ROI at the minimum level. A 15-segments model is indicated for a complete evaluation of the LA deformation, because this model incorporates all available segments, and has the potential to create a real map of the LA electromechanical activation.
